# O01 Trends in antimicrobial resistance amongst pathogens isolated from blood and cerebrospinal fluid cultures in Pakistan (2011–15): a retrospective cross-sectional study

**DOI:** 10.1093/jacamr/dlac003

**Published:** 2022-02-16

**Authors:** Nida Javaid, Qamar Sultana, Karam Rasool, Sumanth Gandra, Fayyaz Ahmad, Safee Ullah Chaudhary, Shaper Mirza

**Affiliations:** Lahore University of Management Sciences, Lahore, Pakistan; Lahore University of Management Sciences, Lahore, Pakistan; Lahore University of Management Sciences, Lahore, Pakistan; Lahore University of Management Sciences, Lahore, Pakistan; Lahore University of Management Sciences, Lahore, Pakistan; Lahore University of Management Sciences, Lahore, Pakistan; Lahore University of Management Sciences, Lahore, Pakistan

## Abstract

**Background:**

While antimicrobial resistance (AMR) continues to be a major public health problem in Pakistan, data regarding trends of resistance among pathogenic bacteria remain scarce, with few studies presenting long-term trends in AMR. This study was therefore designed to analyse long-term AMR trends at a national level in Pakistan.

**Methods:**

We report here results of a comprehensive analysis of resistance among pathogens isolated from blood and CSF, between 2011 and 2015. Susceptibility data were obtained from a local laboratory with collection points all across Pakistan (Chughtai Laboratory). Resistance proportions to most commonly used antimicrobials were calculated for each pathogen over a period of 5 years.

**Results:**

While *Acinetobacter* species demonstrated highest resistance rates to all tested antimicrobials, a sharp increase in carbapenem resistance was the most noticeable (50%–95%) between 2011 and 2015. Our results also highlight the presence of third and fourth generation cephalosporins resistance in *Salmonella enterica* serovar Typhi in Pakistan. Interestingly, where a rise in AMR was being observed in some major invasive pathogens, decreasing resistance trends were observed in *Staphylococcus aureus* to commonly used antimicrobials.

**Conclusions:**

Overall pathogens isolated from blood and CSF between 2011 and 2015 showed an increase in resistance towards commonly used antimicrobials.

